# In-Silico Evidence for a Two Receptor Based Strategy of SARS-CoV-2

**DOI:** 10.3389/fmolb.2021.690655

**Published:** 2021-06-09

**Authors:** Edoardo Milanetti, Mattia Miotto, Lorenzo Di Rienzo, Madhu Nagaraj, Michele Monti, Thaddeus W. Golbek, Giorgio Gosti, Steven J. Roeters, Tobias Weidner, Daniel E. Otzen, Giancarlo Ruocco

**Affiliations:** ^1^ Department of Physics, Sapienza University, Rome, Italy; ^2^ Center for Life Nano and Neuro Science, Italian Institute of Technology, Rome, Italy; ^3^ Interdisciplinary Nanoscience Center (iNANO), Aarhus University, Aarhus, Denmark; ^4^ Centre for Genomic Regulation (CRG), the Barcelona Institute for Science and Technology, Barcelona, Spain; ^5^ RNA System Biology Lab, Department of Neuroscience and Brain Technologies, Istituto Italiano di Tecnologia, Genoa, Italy; ^6^ Department of Chemistry, Aarhus University, Aarhus, Denmark

**Keywords:** sialic acid, SARS-CoV-2, spike (S) protein, shape complementarity, zernike moments

## Abstract

We propose a computational investigation on the interaction mechanisms between SARS-CoV-2 spike protein and possible human cell receptors. In particular, we make use of our newly developed numerical method able to determine efficiently and effectively the relationship of complementarity between portions of protein surfaces. This innovative and general procedure, based on the representation of the molecular isoelectronic density surface in terms of 2D Zernike polynomials, allows the rapid and quantitative assessment of the geometrical shape complementarity between interacting proteins, which was unfeasible with previous methods. Our results indicate that SARS-CoV-2 uses a dual strategy: in addition to the known interaction with angiotensin-converting enzyme 2, the viral spike protein can also interact with sialic-acid receptors of the cells in the upper airways.

## 1 Introduction

At the time of writing, the COVID-19 outbreak represents a serious threat to public health (Huang et al., 2020; Walls et al., 2020; Zhu et al., 2020), and the World Health Organization has declared it a pandemic.

To date, seven coronavirus strains are known to infect humans. In particular, in the past 2 decades, along with SARS-CoV-2, two other β-coronavirus have caused three of the most severe epidemics worldwide: SARS-CoV (Drosten et al., 2003; Ksiazek et al., 2003) and MERS-CoV (Zaki et al., 2012) that respectively cause the severe acute respiratory syndrome (SARS), and the Middle-East respiratory syndrome (MERS).

The characteristics of the interaction between these viruses and human cell receptors are being extensively studied to shed light on both diffusion speed and mortality rate differences between SARS-CoV-2 and the others, with special regard to SARS-CoV.

Indeed, the epidemics of SARS-CoV in 2003 spread across 26 countries on six continents and caused a total of 8,096 cases and 774 deaths (9.6%) (Xu et al., 2020), with an incubation period of 1–4 days (Lessler et al., 2009). On the other side, it has been demonstrated that the latency of SARS-CoV-2 varies from 3–7 days on average, up to 14 days (Zhu et al., 2020). Thus, the average latency of SARS-CoV-2 is slightly longer than that of SARS-CoV (Xu et al., 2020). Moreover, it is estimated from epidemiological data that individuals infected with SARS-CoV-2 are contagious from the beginning of the incubation period and that between the incubation period and the end of the infection each infected individual transmits the infection to about 3.77 other people, according to the first surveys that do not take into account the social-distancing measures that have been imposed in various countries (Yang et al., 2020).

SARS-CoV-2, similarly to SARS-CoV and MERS-CoV, attacks the lower respiratory system, thus causing viral pneumonia. However, this infection can also affect the gastrointestinal system, heart, kidney, liver, and central nervous system (Su et al., 2016; Prompetchara et al., 2020; Zhu et al., 2020). To face the emergency of this pandemic it is essential to reveal the details of the interaction mechanisms between the virus and the human cell receptors. It is well characterized that and how SARS-CoV infection is mediated by the high-affinity interactions between the receptor-binding domain (RBD) of the spike (S) glycoprotein and the human-host angiotensin-converting enzyme 2 (ACE2) receptor (Li F. et al., 2005; Li W. et al., 2005; Li, 2008). The spike protein is located on the virus envelope and enables attachment to the host cell and the fusion between the virus and the cellular membrane. (Kuo et al., 2000; Graham and Baric, 2010).

It has been shown that several critical residues in SARS-CoV-2’s RBD provide favorable interactions with human ACE2, consistent with SARS-CoV-2’s capability to infect the cell (Du et al., 2009; Hoffmann et al., 2020). On the experimental side, it has been confirmed by *in-vivo* experiments that SARS-CoV-2’s entry is mediated by lung-cell ACE2 receptors (Zhou et al., 2020). More importantly, the structure of the spike-ACE2 receptor complex has been determined by cryo-EM (Yan et al., 2020). In conclusion, it is now understood that SARS-CoV-2 binds to the ACE2 receptor to infect the host cell using its spike protein’s RBD, even if it has most likely evolved from SARS-CoV independently (Andersen et al., 2020).

From this point of view, the understanding of the molecular mechanism(s) of the interaction between the ACE2 receptor and the spike protein of the virus can be a key factor designing new drug compounds (Miotto et al., 2021; Di Rienzo et al., 2021). With this aim, computational methods based on both sequence and structure studies of proteins represent a powerful tool (Wu et al., 2020). Indeed, the development of effective computational methods for predicting the binding sites of proteins can improve the understanding of many molecular mechanisms (Kortemme et al., 2004; Donald, 2011; Gainza et al., 2020). Several methods to analyze protein interaction have used protein-surface information (Sharp and Honig, 1990; Duhovny et al., 2002; Shulman-Peleg et al., 2004; Daberdaku and Ferrari, 2019).

Moreover, given the great interest in the structural characterization of interacting regions of proteins (Chen et al., 2003; De Vries et al., 2010; Obarska-Kosinska et al., 2016; Miotto et al., 2020), a wide number of parameter-free methods have been developed. Some of these methods are based on atom densities (Mitchell et al., 2001) or tessellation (Walls and Sternberg, 1992; Li et al., 2007), while others are based on the series exploration of a set of function, such as spherical harmonics (Leicester et al., 1988; Max and Getzoff, 1988), Fourier-correlation theory (Gabb et al., 1997), Wigner D-functions (Saberi Fathi et al., 2014) or 3D Zernike polynomials (Venkatraman et al., 2009; Kihara et al., 2011; Di Rienzo et al., 2017; Daberdaku and Ferrari, 2019; Di Rienzo et al., 2020; Guzenko et al., 2020).

Here, we adopt a recently developed and parameter-free method to efficiently describe the shape of molecular surfaces with the 2D-Zernike formalism (Milanetti et al., 2021).

We apply our formalism to study the interaction between the spike protein and its membrane receptors, comparing SARS-CoV-2 with both SARS-CoV and MERS-CoV. We demonstrate that the actual regions of binding between the spike protein and ACE2 human—both in SARS-CoV and SARS-CoV-2—have a higher complementarity as compared to other randomly sampled exposed receptor regions.

Furthermore, we also analyze in detail the structural properties of the MERS-CoV spike protein that, like several other proteins belonging to the coronavirus family, can interact with sialic acids (Tortorici et al., 2019). Among other coronaviruses, the bovine coronavirus (BCoV), and the two human coronaviruses OC43 and HKU1 are known to bind with N-acetyl-9-O-acetylated sialic-acid (9-O-Ac-Sia) present as terminal residues of glycan chains on glycoproteins and lipids at the cell-surface, acting as cell receptors (Hulswit et al., 2019). This interaction is essential for the initiation of an infection (Schwegmann-Weßels and Herrler, 2006). In particular, we here propose a possible alternative mechanism of SARS-CoV-2 cellular infection, through spike-protein interaction with sialic-acid receptors of the upper airways, similarly to what has been shown for the MERS spike protein (Park et al., 2019). We identify a surface region in the N-terminal domain of the SARS-CoV-2 spike that is very similar to the MERS-CoV spike sialic-acid binding region, and reveal that the spike regions have a comparable charge, which is compatible to sialic acid. Furthermore, this hypervariable region presents several sequence insertions with respect to SARS-CoV that allow the specific residue rearrangement (Zhou et al., 2020). Together, these observations suggest that these MERS-CoV and SARS-CoV-2 regions potentially share an analogous function. This additional cell attachment mechanism of SARS-CoV-2 besides its ACE2 binding, could explain its high diffusion speed.

## 2 Results

In the last decade, the 3D Zernike formalism has been widely applied for the characterization of molecular interactions (Venkatraman et al., 2009; Kihara et al., 2011; Di Rienzo et al., 2017; Daberdaku and Ferrari, 2019).

**FIGURE 1:**
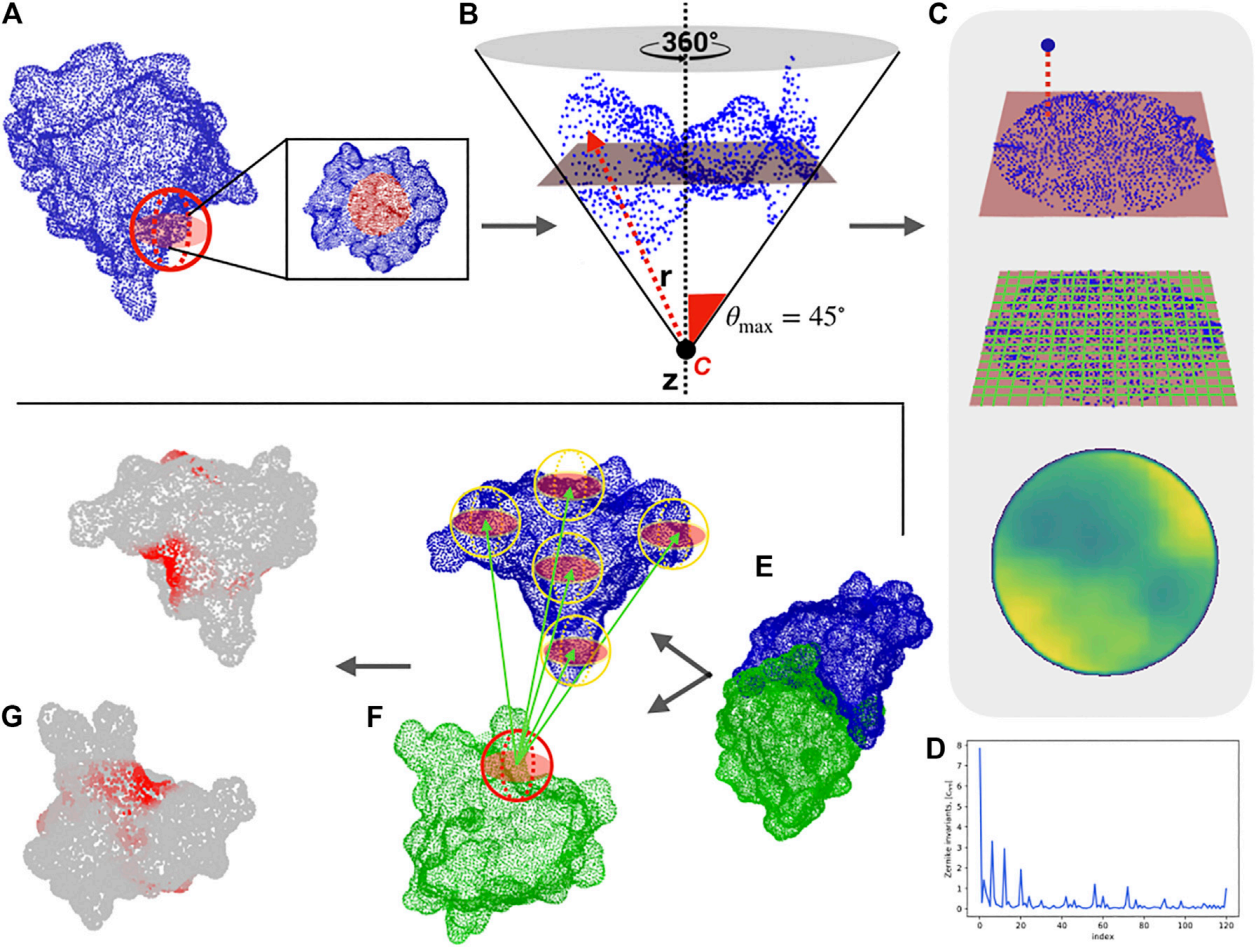
Computational protocol for the characterization of each surface region and the blind identification of the binding sites. **(A)** Molecular solvent-accessible surface of a protein (in blue) and example of patch selection (red sphere). **(B)** The selected patch points are fitted with a plane and reoriented in such a way that the *z*-axis (dotted line) passes through the centroid of the points and is orthogonal to the plane. A point C along the *z*-axis is defined, such as that the largest angle between the perpendicular axis and the secant connecting C to a surface point is equal to 45°. Finally, to each point, its distance, *r* with point C is evaluated. **(C)** Each point of the surface is projected on the fit plane, which is binned with a square grid. To each pixel, the average of the r values of the points inside the pixel is associated. **(D)** The resulting 2D projection of the patch can be represented by a set of 2D Zernike invariant descriptors. **(E**–**F)** Given a protein-protein complex (PDB code: 3B0F, in this example), for each surface vertex we select a patch centered on it and compute its Zernike descriptors. To blindly identify the binding sites, each sampled patch is compared with all the patches of the molecular partner, after which the minimum distance between its patch and all the patches of the molecular partner is associated with each vertex. **(G)** The surface point values are smoothed to highlight the signal in the regions characterized mostly by low distance values, (i.e. high shape complementarity).

To describe portions of molecular surfaces, we adopt a new representation, based on the 2D Zernike polynomials, which allows the quantitative characterization of protein surface regions. As shown in Figure 1, our computational protocol associates an ordered set of numbers (the norm of the expansion coefficients) to each molecular patch, which describes its shape.

Through this compact description, it is possible to both analyze the similarity between two different regions—suggesting, for example, a similar ligand for two binding regions—and to study the complementarity between two interacting surfaces. For a given complex, we select the interacting regions and characterized them with the 2D Zernike invariant descriptors. Therefore, each binding site is associated with a one-dimensional vector, allowing us to easily compare the shape of protein regions with the euclidean distance between their Zernike descriptors. Two regions are complementary when they are characterized by a low distance between their corresponding Zernike vectors (Venkatraman et al., 2009; Di Rienzo et al., 2020).

To test the ability of the method to describe two interacting regions, we use a structural dataset composed of about 4,500 experimentally determined protein-protein complexes, taken from a recent paper that presented a state-of-the-art patch recognition computational method (Gainza et al., 2020). In particular, we first determine the distance decrease of the Zernike descriptors (see Section 4) for a pair of interacting binding sites as compared to the distance between random patches. Our unsupervized method can recognize the binding regions with respect to random patches with an area under the ROC (receiver operating characteristic) curve (AUC) of 0.70 when considering patches of radius 6 Å (see (Milanetti et al., 2021)). Furthermore, the low computational time needed for the calculation of the 2D-Zernike descriptors allows an extensive sampling of the surfaces of a pair of proteins in a complex. Centering a molecular patch on each surface point, we generate for each protein a very high number of Zernike descriptors. Comparing all the patches of the two proteins, we label each surface point with the *binding propensity*, which is the maximum complementarity recorded between the Zernike descriptors of the patch and all the others belonging to the molecular partner surface. The real binding region is expected to be demarcated and mostly composed of elements with high complementarity. To make the binding region’s high complementarity more evident, we smooth the signal by attributing the average value of the vertices closer than 6 Å to each vertex of the surface (see Section 4).

As an example, we report in Figure 1 the protocol of our method for a specific case (PDB code: 3B0F), where this procedure clearly identifies the binding regions of the two proteins (see Section 4 and Ref (Milanetti et al., 2021). for further details). In what follows, we apply the procedure to analyze the interactions of the SARS-CoV-2 spike protein with its membrane receptors in detail, comparing the SARS-CoV-2 spike with the SARS-CoV and MERS-CoV variants.

### 2.1 Comparison Between the Complementarity of the SARS-CoV and SARS-CoV-2 Spike Protein With the Human ACE2 Receptor

**FIGURE 2:**
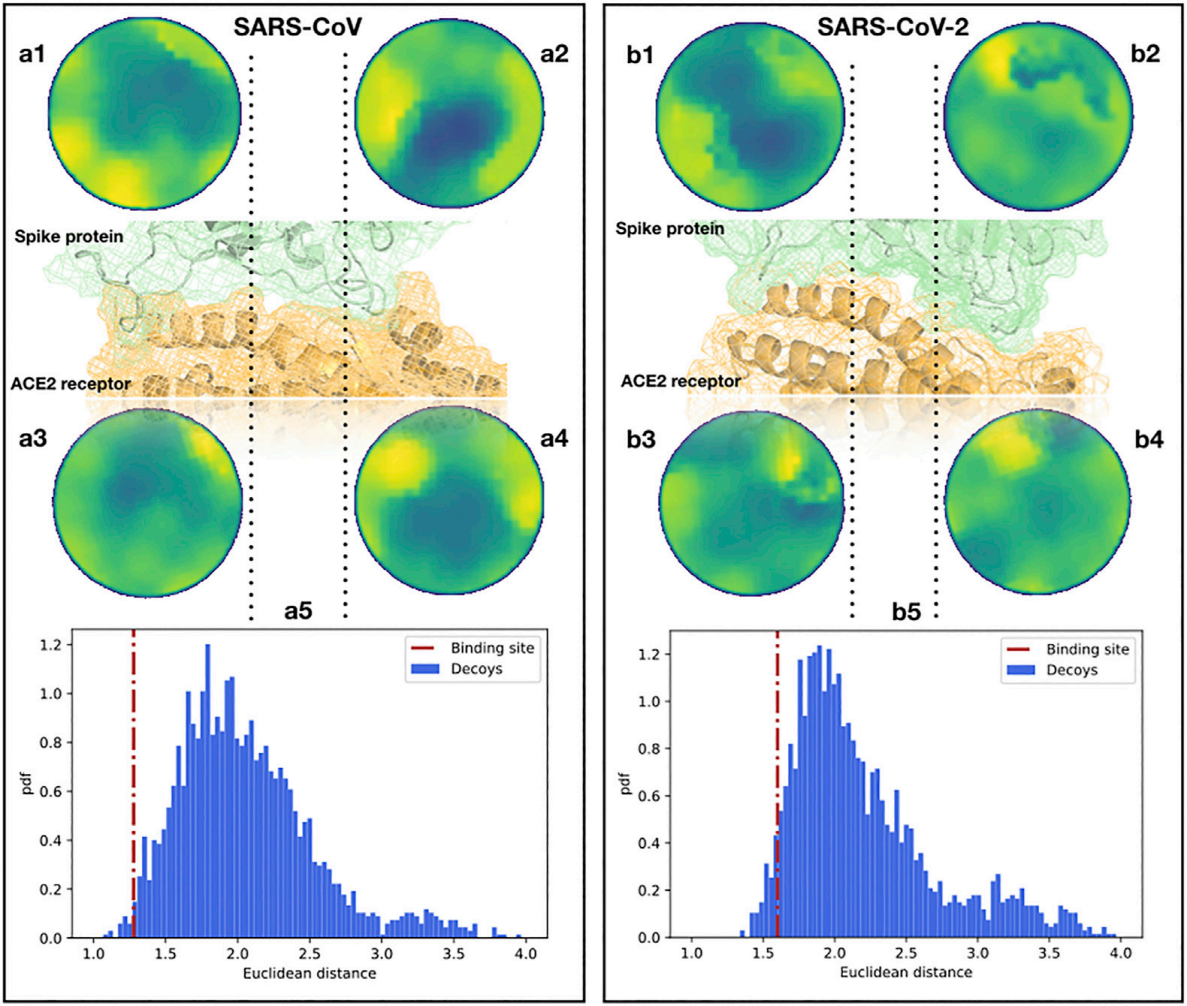
Comparison between the binding regions of the SARS-CoV and SARS-CoV-2 spike protein with human ACE2. **(A1,2)** Patch projections in the unitary circle (see Section 4) for the two ACE2 binding regions of the SARS-CoV spike protein. **(A3,4)** Patch projections in the unitary circle for the SARS-CoV spike binding regions of the human ACE2 receptor. **(A5)** Distance distribution between the two SARS-CoV spike binding sites on ACE2 and randomly selected patches on the spike protein of SARS-CoV. Decoy patches are sampled taking two random regions separated by the same distance measured between the centers of the spike-ACE2 binding site identified in the experimental structure. The red dotted line represents the distance between the real ACE2 and spike patches, calculated from the experimental structure of the complex. **(B)** The same as **(A)** but for the binding site of SARS-CoV-2 and the human ACE2 receptor. The real distances are in the first and fifth percentiles of the distributions for SARS-CoV and SARS-CoV-2, respectively.

To begin with, we analyzed the shape complementarity between the spike proteins of SARS-CoV and SARS-CoV-2 in complex with the human ACE2 receptor (Li F. et al., 2005; Yan et al., 2020). A similar direct comparison can not be performed for other coronaviruses, like MERS-CoV, as those use other cellular receptors. It is interesting that the contact between the spike protein and the ACE2 receptor both for SARS-CoV and SARS-CoV-2 occurs in two spatially distinct interacting regions (see Figure 2), meaning that we need to investigate the two interacting regions separately. When comparing the two Zernike distances (see Section 4), we found that the ACE2-SARS-CoV distance is smaller than the ACE2-SARS-CoV-2 one, but for both complexes the complementarity is much higher than the one would find in other random regions of the complexes (see Figure 2). Note that for an appropriate comparison, we need to define a suitable ensemble of random patches. Indeed, the random regions are sampled from the molecular surface of the spike protein imposing that the distance between the centers of the two patches is similar to the binding region observed in the experimental complex. Then, both the real spike binding region and the ensemble of 1,000 sampled regions are compared with the receptor binding sites.

The results of this analysis are shown in Figure 2. We show the distance distribution of the random regions and we report the distance between the real binding regions, both for the ACE2-SARS-CoV and ACE2-SARS-CoV-2 complex. As the method works in recognizing interacting patches, real binding regions show a higher complementarity (lower distance) than the randomly sampled regions. Furthermore, this analysis shows that the ACE2 receptor has a slightly higher shape complementarity with SARS-CoV than with SARS-CoV-2 spike protein, ∼1.3 vs. ∼1.7, respectively. The results are in line with recent experimental data (Walls et al., 2020).

To validate the stability of the interaction and verify if the interaction patch maintains its molecular surface shape over time, we perform a molecular-dynamics (MD) simulation of the complex consisting of the ACE2 receptor and the spike protein of SARS-CoV-2 (see Section 4 section for details). As we show in Supplementary Figure S1 of the Supporting Information, a comparison between ACE2 patches at different times of the equilibrium MD simulation gives a constant Zernike distance value of ∼1, which set to ∼1 the lower bound of thermal noise for this system. In fact, the MD analysis allows us to quantify, at least for this system, the difference between the Zernike descriptors associated with different conformations that the same patch explores due to thermal fluctuations.

### 2.2 Identification of Another Possible Binding Region of the SARS-CoV-2 Spike

Although it is currently known that the spike protein of SARS-CoV-2 binds to the ACE2 receptor of host cells (Hoffmann et al., 2020; Wan et al., 2020), the investigation of possible other infection mechanisms is important in the study of this disease. Specifically, in ref. (Zhou et al., 2020), the authors underline the necessity to elucidate whether SARS-CoV-2 spike protein could have acquired the ability to bind with sialic acid as MERS-CoV does. Indeed, it has been recently shown that besides the usual receptor (dipeptidyl-peptidase four receptor), MERS-CoV spike protein interacts with sialic-acid molecules (Park et al., 2019) using a well-identified pocket in the N-terminal region of the protein. This makes the virus able to interact with the upper airways and subsequently reach the lower-airway cells (Li et al., 2017). The recognition between the MERS-CoV spike protein and sialic-acids molecules occurs via a conserved groove that plays a key role in MERS-CoV spike mediated attachment to sialosides and subsequent entry into human airway epithelial cells (Park et al., 2019).

Since the interaction of the MERS-CoV spike and the sialic-acids is caused mainly by hydrogen bonds and shape complementarity (Tortorici et al., 2019), our method is particularly suitable to find a region on the SARS-CoV-2 spike surface that is similar to the one involved in sialic-acid binding by the MERS-CoV spike. Using the experimental structure of the MERS-CoV spike in complex with sialic-acid molecule (Park et al., 2019), we extracted its binding region and we described it with Zernike descriptors. Then, we sampled the corresponding domains of both SARS-CoV and SARS-CoV-2 spike, building a molecular patch on each surface point and characterizing it with its corresponding Zernike descriptors. Each region sampled from the spike proteins of these two viruses is then compared with the MERS-CoV spike binding region, looking for a similar region that can mediate interaction with a similar ligand.

**FIGURE 3:**
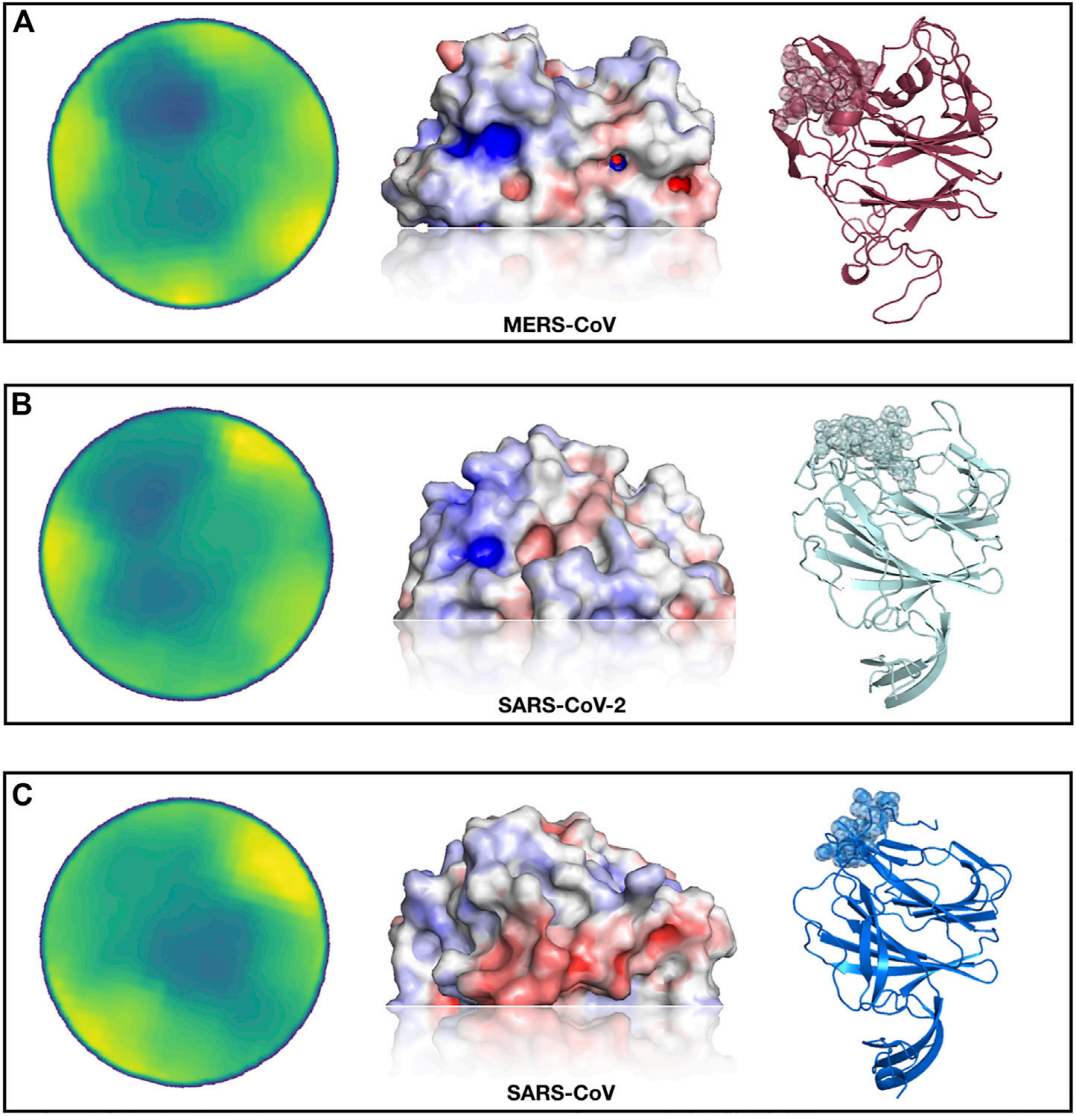
Identification of a SARS-CoV-2 spike region very similar to the sialic-acid binding site on MERS-CoV spike. **(A)** From left to right, projected region of the real sialic-acid binding site on MERS-CoV, electrostatic potential surface of the same region and cartoon representation of the MERS-CoV spike protein with the binding site highlighted. **(B)** Putative sialic-acid binding region on SARS-CoV-2 as predicted by our Zernike-based method. From left to right, the projected region of putative interaction site between SARS-CoV and sialic acid, electrostatic potential surface, and cartoon representation of the SARS-CoV spike protein with the binding site highlighted. **(C)** Same as **(B)** but for SARS-CoV spike protein.

In Figure 3, we show the results of this analysis. In particular, selecting the region most similar to the MERS-CoV binding site, we identified—both for the SARS-CoV and SARS-CoV-2 spike protein—one region with a high resemblance to the sialic-acid binding region of the MERS-CoV spike.

Interestingly, the best region of the SARS-CoV-2 spike exhibits a higher similarity than the pocket selected by the SARS-CoV spike. We moreover calculate the electrostatic potential of the involved surfaces with the eF-surf web-server (Kinoshita and Nakamura, 2004). As shown in Figure 3, in cartoon representation, the region found in the molecular surface of the SARS-CoV-2 spike is very similar to the MERS spike region that interacts with sialic-acid, both in terms of electrostatic potential and in shape. However, the region identified on the SARS-CoV spike exhibits an electrostatic configuration very dissimilar from the sialic-binding site in the MERS-CoV spike, which makes the interaction with sialic-acid in that region very unlikely.

**FIGURE 4:**
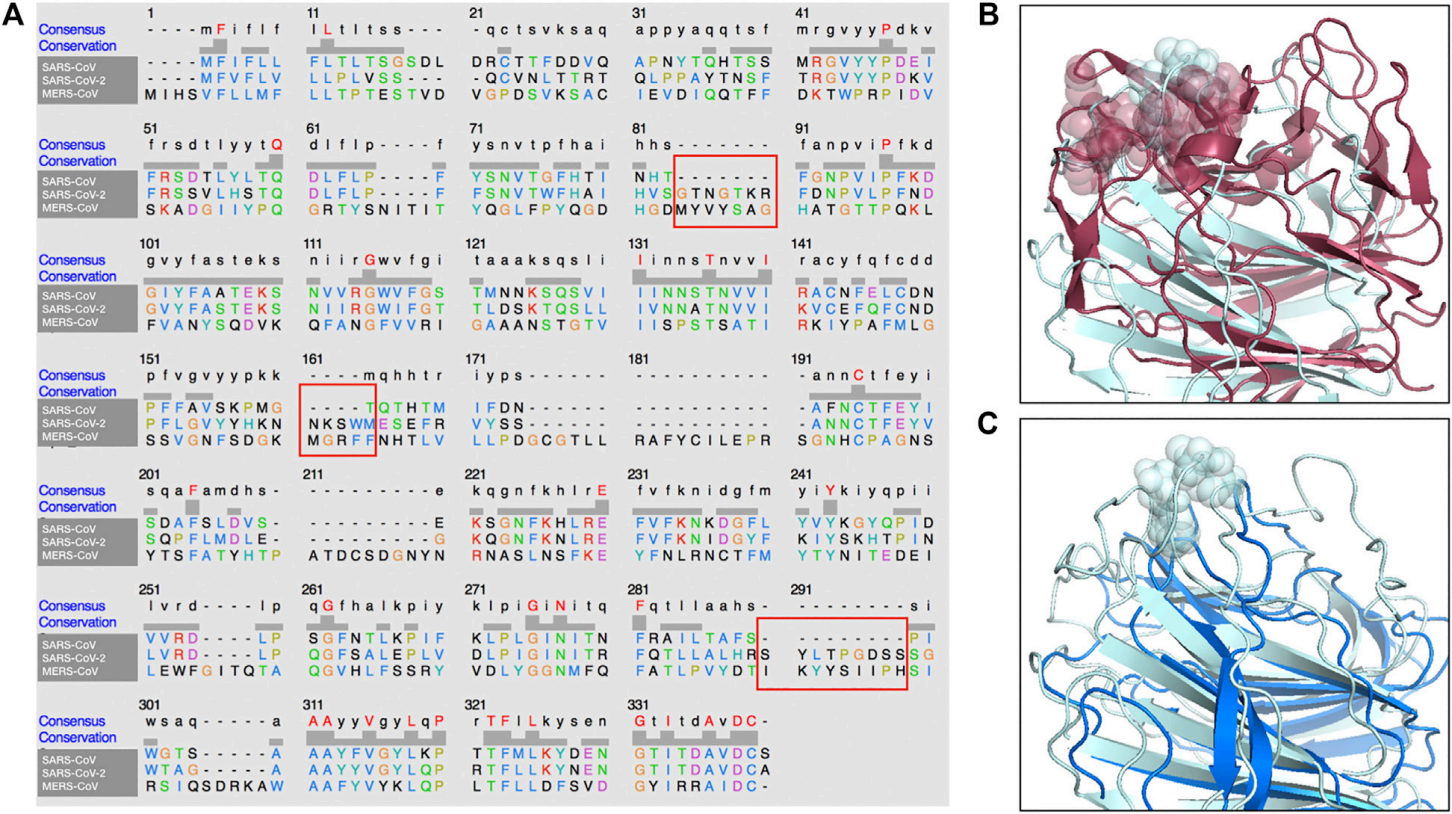
Sequence and structure comparison of the N-terminal region of MERS-CoV, SARS-CoV-2 and SARS-CoV. **(A)** A multiple sequence alignment between the MERS-CoV, the SARS-CoV and the SARS-CoV-2 spike protein sequence. **(B)** Structural comparison between MERS-CoV and SARS-CoV-2 A-domain. The three segments of the sialic-acid binding site for MERS-CoV spike and the proposed binding site on SARS-CoV-2 spike are highlighted. **(C)** Structural comparison between SARS-CoV and SARS-CoV-2 A-domain. The proposed binding site on SARS-CoV-2 has no corresponding structure in the SARS-CoV spike.

In addition, in Figure 4, we present a multiple sequence alignment—with software Clustal Omega (Sievers et al., 2011)—between the three spike proteins, in order to highlight the position of the insertions found in SARS-CoV-2 spike with respect to SARS-CoV.

Importantly, the proposed sialic-acid binding site on the SARS-CoV-2 spike, besides being structurally in a surface region bordering the corresponding MERS-CoV pocket, is composed of a set of consecutive residues (residue number 73–76) constituting an insertion in respect to the SARS-CoV spike sequence. Thus, this insertion in the N-terminal domain of the spike protein could confer the capability of infecting human cells in a dual strategy to SARS-CoV-2, which results in the high diffusion speed of this new virus.

## 3 Discussions and Conclusion

A blind prediction of the interaction regions between molecules is still an open challenge, despite the great steps that have been made. However, the need for fast and reliable theoretical and computational tools, capable to guide and speed-up experiments, becomes especially important when we face crizes like the present one. Despite the great efforts to contain it, both in terms of public policies and scientific research, the human infection caused by the novel SARS-CoV-2 is still spreading at an impressive rate, and the pandemic is far from being under control. During the last months extensive studies have been published about the virus-host interactions focusing in particular on the various stages of the cell internalization mechanism. Several works found that, in analogy with the case of SARS-CoV, SARS-CoV-2 uses its spike protein to bind to ACE2 receptors, most expressed in the lower respiratory ways. Further experimental investigations revealed a comparable receptor-binding affinity between the novel coronavirus and the older SARS-CoV, even if the binding regions display a certain degree of variability (Andersen et al., 2020). The modest difference in binding affinity seems insufficient to explain the higher human-human transmission rate with respect to SARS-CoV and the overall sequence variability suggests that SARS-CoV-2 may have optimized in other directions, such as in acquiring the ability to bind to other receptors (Zhou et al., 2020).

In this work, we adopt a new fast computational method that compactly summarizes the morphological properties of a surface region of a protein. Testing the unsupervized method on a large dataset of protein-protein interactions, we proved its ability to correctly recognize the high shape complementarity occurring between interacting surfaces. Analyzing the available experimental structures of SARS-CoV-2 Spike protein in complex with human ACE2, we found that the binding region presents indeed a comparable (slightly lower) shape complementarity with the analogous complex of SARS-CoV. Such a minimal difference enforces the hypothesis that the apparent higher fitness of SARS-CoV-2 lies elsewhere.

In particular, looking at other members of the large coronavirus family, one finds that many members developed the ability to bind to two distinct receptors, with one binding site in the C-terminal domain of the S-protein that generally binds protein-like receptors (like ACE2 for SARS-CoV and SARS-CoV-2) and the other situated in the N-terminal region, usually able to bind to sugar-like receptors. In particular, MERS-CoV has been found able to bind to sialic-acid receptors both in camel, human, and bat cells. Applying our method to the sialic-acid binding region, which has been recently determined experimentally in MERS-CoV, we have found an exceptionally similar region in the corresponding region of the SARS-CoV-2 spike. This region, similar in structure to the MERS-CoV corresponding one and absent in SARS-CoV (see Figure 4), could be able to mediate a low-affinity, but high-avidity interaction with sialic acids. Interestingly, the sequence variability of the spike protein, recently determined for the SARS-CoV-2 sequences of 62 different strains (Vandelli et al., 2020), shows a high conservation level of the ACE2 binding site while the highest variability is located in the region that we indicate here to be potentially involved in sialic-acid biding: this confirms the importance of this region in regulating host-cell infection (Qing et al., 2020).

Finally, while our manuscript was in preparation, an external experimental validation of our prediction has been found In fact, a recent work tested the capability of the spike protein to bind to neuraminic acid using a glyconanoparticle platform (Baker et al., 2020). Indeed, the authors observed a stable binding, demonstrating the SARS-CoV-2 spike glycan-binding function. In addition, the binding site, the authors propose, is in agreement with the region predicted by our fully computational method.

In conclusion, we propose that this dual cell-entry mechanism can explain the high diffusion speed this virus exhibits and we strongly encourage a more accurate investigation into this observation.

## 4 Methods

### 4.1 Experimental Protein Structures

• Complex between SARS-CoV spike protein and human ACE receptor: PDB code 6ACJ.
• Complex between SARS-CoV-2 spike protein and human ACE receptor: PDB code 6M17.
• Complex between MERS spike protein and sialic acid: PDB code 6Q07.
• Unbound SARS-CoV spike protein: PDB code 6CRV.
• Unbound SARS-CoV-2 spike protein: modeled by I-TASSER server (Yang and Zhang, 2015).

#### 4.1.1 Computation of Molecular Surfaces

We use DMS (Richards, 1977) to compute the solvent accessible surface for all proteins structure, given their x-ray structure in PDB format (Berman et al., 2003), using a density of five points per Å^2^ and a water probe radius of 1.4 Å. The unit normals, for each point of the surface, were calculated using the flag −n.

#### 4.1.2 Patch Selection and Space Reduction

Given a molecular surface described as a set of points in a three-dimensional Cartesian space, and a region of interest on this surface, we define a surface patch, ∑, as the intersection of the region of interest and the surface. In principle, the region of interest can have an arbitrary shape, in this work we chose to use a spherical region having radius $R_{s}=6\text{Å}$ and one point of the surface as the center (see Figure 1A). Once the patch is selected, we fit a plane that passes through the points in ∑, and we reorient the patch in such a way that the *z*-axis is perpendicular to the plane and such that it goes through the center of the plane. Then given a point *C* on the *z*-axis we define the angle θ as the largest angle between the perpendicular axis and a secant connecting *C* to any point of the surface ∑. *C* is then set such that $\text{θ}=45^{\circ }$. *r* is the distance between *C* and a surface point. We then build a square grid, and we associate each pixel with the mean *r* of the points inside it. This 2D function can be expanded in the basis of the Zernike polynomials: the norm of the coefficients of this expansion constitute the Zernike invariant descriptors, which are invariant under rotation in space. In the next subsection, we provide a brief summary of the main features of the Zernike basis. There are several good reviews, like (Lakshminarayanan and Fleck, 2011) that offer more detailed discussions. A schematic representation of the procedure is shown in Figures 1B–D.

### 4.1.3 2D Zernike Polynomials and Invariants

Given a function f(r,ϕ) (polar coordinates) defined inside the region r<1 (unitary circle), it is possible to represent the function in the Zernike basis as

$$f\left(r,\phi \right)=\sum_{n=0}^{\infty }\sum_{m=0}^{m=n}c_{nm}Z_{nm}\left(r,\phi \right),$$ (1)

with

$$c_{nm}=\frac{\left(n+1\right)}{\pi }\langle Z_{nm}|f\rangle =\frac{\left(n+1\right)}{\pi }\int_{0}^{1}drr\int_{0}^{2\pi }d\phi Z_{nm}^{*}\left(r,\phi \right)f\left(r,\phi \right).$$ (2)

being the expansion coefficients. The Zernike polynomials are complex functions, composed by a radial and an angular part,

$$Z_{nm}=R_{nm}\left(r\right)e^{im\phi },$$ (3)

where the radial part for a certain couple of indexes, *n* and *m*, is given by

$$R_{nm}\left(r\right)=\sum_{k=0}^{\frac{n-m}{2}}\frac{{\left(-1\right)}^{k}\left(n-k\right)!}{k!\left(\frac{n+m}{2}-k\right)!\left(\frac{n-m}{2}-k\right)!}r^{n-2k},$$ (4)

In general, for each couple of polynomials, one finds that

$$\langle Z_{nm}|Z_{n^{\prime }m^{\prime }}\rangle =\frac{\pi }{\left(n+1\right)}\delta_{nn^{\prime }}\delta_{mm^{\prime }},$$ (5)

which ensures that the polynomials can form a basis and knowing the set of complex coefficients, $\left\{c_{nm}\right\}$ allows for a univocal reconstruction of the original image (with a resolution that depends on the order of expansion, N=max(n)). We found that N=20, which corresponds to a number of coefficients of 121, allows for a good visual reconstruction of the original image.

By taking the modulus of each coefficient $\left(z_{nm}=\left|c_{nm}\right|\right)$, a set of descriptors can be obtained which have the remarkable feature of being invariant for rotations around the origin of the unitary circle.

The shape similarity between two patches can then be assessed by comparing the Zernike invariants of their associated 2D projections. In particular, the similarity between patch *i* and *j* is measured as the Euclidean distance between the invariant vectors, i.e.

$$d_{ij}=\sqrt{\sum_{k=1}^{M=121}{\left(z_{i}^{k}-z_{j}^{k}\right)}^{2}}.$$ (6)

### 4.1.4 Evaluation of Similarity and Complementarity

When comparing patches, the relative orientation of the patches before the projection in the unitary circle must be evaluated. Intuitively, if we search for similar regions we must compare patches that have the same orientation once projected in the 2D plane, i.e., the solvent-exposed part of the surface must be oriented in the same direction for both patches, for example as the positive *z*-axis. If instead, we want to assess the complementarity between two patches, we must orient the patches contrariwise, i.e., one patch with the solvent-exposed part toward the positive *z*-axis (“up”) and the other toward the negative *z*-axis (“down”).

### 4.1.5 Blind Search of Binding Sites

The velocity of the procedure that produces the set of invariant descriptors from a patch in the 3D surface allows for a vast screening of pairs of surfaces to look for both similar and also complementary regions. In order to identify the binding region between two proteins, a vector of Zernike invariants associated to the “up” patch with that point as its center and another set of invariants to each point of the other surface (in a “down” orientation) is associated to each point of one of the surfaces. Then for each point *i* of say, protein 1, we can compute the Euclidean distance with all the points of the other surface associated with protein two and associate the minimum distance to point *i*, and vice-versa for protein 2 (see Figures 1E–F). A smoothing process of the surface point values is applied in order to highlight the signal in the regions characterized mostly by low distance values, (i.e. high shape complementarity).

### 4.1.6 Molecular Dynamics Simulation

Starting from the x-ray structure of the complex (PDB id:6M17) we performed a 100 ns long simulation with a time step of 2 fs. The system was rendered electroneutral adding 24 sodium counter-ions, with a water density of 998 kg/m^3^. The simulation was performed using Gromacs 2019.3 (Van Der Spoel et al., 2005). Topology of the system was built using the CHARMM-27 force field (Brooks et al., 2009). The protein was placed in a dodecahedric simulative box, with periodic boundary conditions, filled with TIP3P water molecules (Jorgensen et al., 1983). We checked that each atom of the proteins was at least at a distance of 1.1 nm from the box borders. The system was then minimized with the steepest descent algorithm. Next, a relaxation of water molecules and thermalization of the system was run in NVT and NPT environments each for 0.1 ns at 2 fs time-step. The temperature was kept constant at 300 K with the v-rescale algorithm (Bussi et al., 2007); the final pressure was fixed at 1 bar with the Parrinello-Rahman algorithm (Parrinello and Rahman, 1980). The LINCS algorithm (Hess et al., 1997) was used to constraint h-bonds. A cut-off of 12 Å was imposed to evaluate the short-range non-bonded interactions and the Particle Mesh Ewald method (Cheatham et al., 1995) was employed for the long-range electrostatic interactions.

## Data Availability

The original contributions presented in the study are included in the article/Supplementary Material, further inquiries can be directed to the corresponding authors.
