# Supplementary material for: In-Silico Evidence for a Two Receptor Based Strategy of SARS-CoV-2
Source: Front Mol Biosci. 2021 Jun 9;8:690655. doi: 10.3389/fmolb.2021.690655 (PMC8219949; doi:10.3389/fmolb.2021.690655)
Supplement: Supplementary file 1 [file DataSheet1.pdf]

Supporting Information:
In-Silico evidence for a two receptors based strategy of SARS-CoV-2

Edoardo Milanetti*†,[1,2] Mattia Miotto*,[1,2] Lorenzo Di Rienzo*,[2] Madhu Nagaraj*,[3] Michele Monti,[4,5] Thaddeus W. Golbeck,[6] Giorgio Gosti,[2] Steven J. Roeters‡,[6] Tobias Weidner,[6] Daniel Otzen,[3] and Giancarlo Ruocco[1,2]

[1]Department of Physics, Sapienza University, Piazzale Aldo Moro 5, 00185, Rome, Italy
[2]Center for Life Nanoscience, Istituto Italiano di Tecnologia, Viale Regina Elena 291, 00161, Rome, Italy
[3]Interdisciplinary Nanoscience Center (iNANO), Aarhus University, Gustav Wieds Vej 14, 8000 Aarhus C, Denmark
[4]Centre for Genomic Regulation (CRG), The Barcelona Institute for Science and Technology, Dr. Aiguader 88, 08003 Barcelona
[5]RNA System Biology Lab, Department of Neuroscience and Brain Technologies, Istituto Italiano di Tecnologia, Via Morego 30, 16163, Genoa, Italy
[6]Department of Chemistry, Aarhus University, Langelandsgade 140, 8000, Aarhus C, Denmark

---

* These authors contributed equally to the present work.
† Corresponding author: edoardo.milanetti@uniroma1.it
‡ Corresponding author: s.j.roeters@chem.au.dk

I. MOMENTS-BASED DESCRIPTORS

In this section, we report the main properties of some of the most important moments-based shape descriptors. This kind of descriptors was originally developed for image retrieval, and are characterized by different invariance properties that we summarized in the following table. We focus on size invariance, translation invariance, rotation invariance and orthogonality. As shown in Table I, the invariance for rotation and translation makes the Zernike and pseudo-Zernike descriptors very suitable for a fast and absolute comparison of protein regions that belong to different proteins, because they remove the preliminary requirement of structural alignment. Moreover, the orthogonality of the polynomial basis ensures that the information carried out by each descriptor is not redundant [1]. According to the literature [2, 3], we can conclude that, in terms of description accuracy and noise resistance, Zernike-based shape descriptors have demonstrated their superiority over other moment functions.

II. A BRIEF BACKGROUND ON IMAGE MOMENTS

Originally, Hu showed how to drop certain central moments, and mathematically combined the others to form rotationally invariant moments [4, 5]. Unfortunately, as for central moments, these moments are not orthogonal and shape information is lost because many moments are dropped. Later on, various attempts were made to develop orthogonal moments as shape descriptors, the reason is that raw, central, and Hu moments do not form an orthogonal basis, and for this reason, they are correlated. Correlation between moments implies that the moments present redundant information, and for this reason are not optimal descriptors [1]. Consequently, Legendre, Zemike, and pseudo-Zemike moments were developed [1, 6]. Later Revaud et al. [7] showed how phase information could be preserved together with the moment information to improve the effectiveness of Zernike based descriptor as shape descriptors. Furthermore, Krawtchouk and Hahn moments improve on discretization problems because otherwise continuous functions are used as moments of discrete 2D images, unfortunately, these moments are not naturally rotational invariant, and to derive rotational invariance moments several moments need to be dropped resulting in information lose as for Hu moments. Other works generalized moment descriptors to higher dimensions [8, 9]. Thus Zernike and Pseudo-Zernike moments are the only moments to be orthogonal while being rotationally and translationally invariant but preserving scale information. Furthermore, several research papers analyzed the performance of Zernike and Pseudo-Zernike methods as shape descriptors, and found that if these moments were not the best they were second-best in almost all applications [1–3, 6, 7, 10, 11]. For the performance in various comparison test, the invariances and orthogonality properties we decided to use Zernike moments.

A. Comparison between 3D and 2D Zernike moments

Zernike moments formalism may be defined as 2D or 3D. Thus since patches are 3D portions of surfaces that are 2D objects they can be either represented as a 3D object with 3D Zernike moments or as a function defined over a 2D domain with 2D Zernike moments. The advantages and disadvantages of 2D or 3D moments need to be weighted against the kind of performances that we want to get out of 2D Zernike descriptors. Clearly 2D Zernike descriptors require the storage of fewer information and are faster to compute. The advantages of using 3D moments are two. First, even if a surface in a 3D space is a 2D object, to calculate the 2D Zernike moments of every single patch we have to project the patch points on a 2D surface. This 2D projection loses a part of the 3D shape information of the object deforming the relative distances between the point on the surface [8]. Similarly, to what happens when we draw a map of the globe, and depending on the projection that we chose the distances between continents and their areas are deformed. This is a problem for the 2D Zernike representation, nevertheless, it is mitigated as the patches are made smaller and their curvature is reduced. Furthermore, two complementary patches centered in their center of mass will similarly deform their surfaces, thus the effect of deformation is balanced. Second, in 2D Zernike moments we can not fully represent protein iso-surfaces that in the reference polar coordinates are non-injective. Similarly for the projection as the patches became smaller some of the non-injective regions disappear.

From a different perspective, the disadvantage of 3D moments is that the surface of a 3D object is a 2D object, and consequently in 3D the surface is a discontinuous function, in which the points that belong the protein surface have value 1, and the points outside the surface have value 0. If we continuously move from inside to outside the surface we jump from 1 to 0. Consequently, 3D Zernike has to expand a discontinuous function. Thus to reconstruct the original 3D object we have to set a threshold. Moreover, the discontinuity increases the weight of higher-order moments [1].

When we consider 2D Zerniche moments we find that the surface is 2D, and if it is injective it can be expanded with an infinite expansion that can be used to perfectly reconstructed the surface shape. Thus we have to keep in mind that as the patches became smaller the non-injective portion disappears and the distortions caused by the projection

	Size Inv.[1]	Traslation Inv.	Rotation Inv.	Orthogonalily
Raw	No	No	No	No
Central	No	Yes	No	No
Hu	Yes	Yes	Yes	No
Krawtchouk	No[2]	Yes[2]	No[2]	Yes
Hahn	No[2]	Yes[2]	No[2]	Yes
Legendre	No[2]	Yes[2]	No[2]	Yes
Zernike	No	Yes	Yes	Yes
Pseudo-Zernike	No	Yes	Yes	Yes

TABLE I: Table presenting the main invariances and orthogonality of the moments that we are studying.
[1] The size invariance column is set to gray because to understand if two corresponding patches match we do not want the moments to be size invariant.
[2] We can derive rationally invariant moments with Krawtchouk and Legendre with a similar process to the process used to derive Hu moments. Unfortunately this process greatly reduces the information because orthogonal basis must be drooped.

are mitigated. Furthermore, the 2D projection is a continuous function thus higher-order moments are only caused by sampling noise [1].

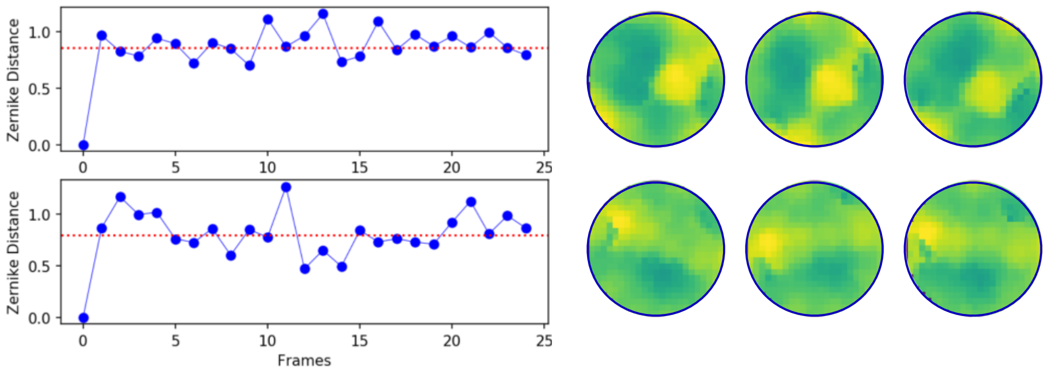

FIG. 1: **Comparison of the Zernike coefficients of the ACE2 binding regions for different frames extracted after thermalization.** Each point corresponds to the Euclidean distance between the Zernike descriptors of a frame with respect to frame 0.

B. Molecular dynamics simulations of the ACE2 - SARS-CoV-2 complex

Simulation was performed using Gromacs 2019.3 [12]. Topologies of the system were built using the CHARMM-27 force field [13]. The protein was placed in a dodecahedric simulative box, with periodic boundary conditions, filled with TIP3P water molecules [14]. For all simulated systems, we checked that each atom of the proteins was at least at a distance of 1.1 nm from the box borders. Each system was then minimized with the steepest descent algorithm. Next, a relaxation of water molecules and thermalization of the system were run in NVT and NPT environments each for 0.1 ns at 2 fs time-step. The temperature was kept constant at 300 K with v-rescale algorithm[15]; the final pressure was fixed at 1 bar with the Parrinello-Rahman algorithm[16] LINCS algorithm[17] was used to constraint h-bonds. while a cut-off of 12 $\mathring{A}$ was imposed for the evaluation of short-range non-bonded interactions and the Particle Mesh Ewald method [18] for the long-range electrostatic interactions. The described procedure was used for all the performed simulations. In the following, we provide further details, specific of each simulation.

Starting from the x-ray structure of the complex (PDB id:6M17) we performed a 100 ns long simulation with time step 2 fs. The system was rendered electroneutral adding 24 sodium counterions, with a water density of 998 kg/m^3.

[1] C. H. Teh and R. T. Chin, IEEE Transactions on Pattern Analysis and Machine Intelligence **10**, 496 (1988), ISSN 01628828.
[2] S. O. Belkasim, M. Shridhar, and M. Ahmadi, Pattern recognition **24**, 1117 (1991).
[3] S. X. Liao and M. Pawlak, IEEE Transactions on Pattern Analysis and Machine Intelligence **18**, 254 (1996).
[4] M. K. Hu, IRE Transactions on Information Theory **8**, 179 (1962), ISSN 21682712.
[5] J. Flusser and T. Suk, IEEE Transactions on Image Processing **15**, 3784 (2006), ISSN 10577149.
[6] M. R. Teague, Journal of the Optical Society of America **70**, 920 (1980), ISSN 0030-3941, URL `https://www.osapublishing.org/viewmedia.cfm?uri=josa-70-8-920{\&}seq=0{\&}html=truehttps://www.osapublishing.org/abstract.cfm?uri=josa-70-8-920https://www.osapublishing.org/josa/abstract.cfm?uri=josa-70-8-920https://www.osapublishing.org/abstract.cfm?URI=josa-70-8-920`.
[7] J. Revaud, G. Lavoué, and A. Baskurt, IEEE Transactions on Pattern Analysis and Machine Intelligence **31**, 627 (2009), ISSN 01628828, URL `https://pubmed.ncbi.nlm.nih.gov/19229079/`.
[8] R. Chikhi, L. Sael, and D. Kihara, Proteins: Structure, Function, and Bioinformatics **78**, 2007 (2010), ISSN 08873585, URL `http://doi.wiley.com/10.1002/prot.22715`.
[9] S. M. Saberi Fathi, D. T. White, and J. A. Tuszynski, Proteins: Structure, Function, and Bioinformatics **82**, 2756 (2014).
[10] P. T. Yap, R. Paramesran, and S. H. Ong, IEEE Transactions on Image Processing **12**, 1367 (2003), ISSN 10577149.
[11] P. T. Yap, R. Paramesran, and S. H. Ong, IEEE Transactions on Pattern Analysis and Machine Intelligence **29**, 2057 (2007), ISSN 01628828.
[12] D. V. D. Spoel, E. Lindahl, B. Hess, G. Groenhof, A. E. Mark, and H. J. C. Berendsen, Journal of Computational Chemistry **26**, 1701 (2005).
[13] B. R. Brooks, C. L. Brooks, A. D. Mackerell, L. Nilsson, R. J. Petrella, B. Roux, Y. Won, G. Archontis, C. Bartels, S. Boresch, et al., Journal of Computational Chemistry **30**, 1545 (2009).
[14] W. L. Jorgensen, J. Chandrasekhar, J. D. Madura, R. W. Impey, and M. L. Klein, The Journal of Chemical Physics **79**, 926 (1983).
[15] G. Bussi, D. Donadio, and M. Parrinello, The Journal of Chemical Physics **126**, 014101 (2007).
[16] M. Parrinello and A. Rahman, Physical Review Letters **45**, 1196 (1980).
[17] B. Hess, H. Bekker, H. J. C. Berendsen, and J. G. E. M. Fraaije, Journal of Computational Chemistry **18**, 1463 (1997).
[18] T. E. I. Cheatham, J. L. Miller, T. Fox, T. A. Darden, and P. A. Kollman, Journal of the American Chemical Society **117**, 4193 (1995).
